# A neuromorphic electronic artist for robotic painting

**DOI:** 10.1038/s41598-025-92081-x

**Published:** 2025-06-04

**Authors:** Lioba Schürmann, Giulia D’Angelo, Liat Grayver, Chiara Bartolozzi, Giacomo Indiveri

**Affiliations:** 1 Institute of Neuroinformatics, University of Zurich and ETH, Zurich, Switzerland; 2 Event-Driven Perception for Robotics, Italian Institute of Technology, Genoa, Italy; 3 Department of Cybernetics, Faculty of Electrical Engineering, Czech Technical University, Prague, Czech Republic; 4 Collegium Helveticum, Zurich, Switzerland

**Keywords:** Electrical and electronic engineering, Computer science

## Abstract

Recent advances in deep learning have sparked interest in AI-generated art, including robot-assisted painting. Traditional painting machines use static images and offline processing without considering the dynamic nature of painting. Neuromorphic cameras, which capture light intensity changes through asynchronous events, and mixed-signal neuromorphic processors, which implement biologically plausible spiking neural networks, offer a promising alternative. In this work, we present a robotic painting system comprising a 6-DOF robotic arm, event-based input from a Dynamic Vision Sensor (DVS) camera and a neuromorphic processor to produce dynamic brushstrokes, and tactile feedback from a force-torque sensor to compensate for brush deformation. The system receives DVS events representing the desired brushstroke trajectory and maps these events onto the processor’s neurons to compute joint velocities in close-loop. The variability in the input’s noisy event streams and the processor’s analog circuits reproduces the heterogeneity of human brushstrokes. Tested in a real-world setting, the system successfully generated diverse physical brushstrokes. This network marks a first step towards a fully spiking robotic controller with ultra-low latency responsiveness, applicable to any robotic task requiring real-time closed-loop adaptive control.

## Introduction

The recent surge in AI-generated art promises to enhance artistic capabilities and foster entirely new forms of creative expression beyond human limitations. While the majority of research has focused on the generation of digital images, such as OpenAI’s DALL-E model^1^, there has not been much work conducted on physical painting machines which face additional challenges. These challenges include the need to master complex materials and tools such as paper, paint, and brushes, and to produce brushstrokes that exhibit the same complexity, unique variability, and traits as those created by humans. To address this, it is essential to gain an understanding of the intrinsic characteristics of human brushstrokes and the physical interaction of all the elements that contribute to the final visual effect. At the same time, it is vital to create a robotic control system that can reproduce the variability and heterogeneity of human brushstrokes in a natural and faithful manner. This project bridges these elements by developing an adaptive artificial robotic sensory-processing motor system that incorporates principles of traditional craftsmanship to facilitate the generation of robotic-based paintings. The creation of robotic artworks relies on recreating the painting experience by generating a sequence of actions to reconstruct a desired drawing from a human. A major challenge is the translation of the simulated brushstroke plan into the physical actions of the robot. Some painting systems address this challenge by minimizing the disparity between their simulated planning and the real environment and by relying on equipment with known models, such as the E-DAVID robot^2^. The FRIDA robot employs a brushstroke model comprising the length, bend and thickness of the stroke and learns a mapping between these parameters and the stroke’s appearance from real data^3^. Previous painting systems also differ in whether they incorporate visual feedback into their painting process. Systems like the Dark Factory portraits execute their painting plans in open-loop^4^, whereas other systems, such as the FRIDA robot, incorporate visual feedback and iteratively re-optimise their painting plan in closed-loop after adding several new brushstrokes at a time.

One limitation of existing painting machines is their reliance on digital controllers that are bit-precise and 100% reproducible. The complexity of the painting brush introduces some degree of noise into the painting process, but the resulting creations are often homogeneous and lack human-like features.

In contrast to digital controllers, subthreshold, mixed-mode, neuromorphic circuits aim to model the structure and computational principles of the nervous system and produce brain-like robust computation on hardware that exhibits limitations similar to those of the biological nervous system, including noise, mismatch, thermal fluctuations, limited precision, and restrictions in connectivity and wiring. As such, neuromorphic systems are likely to produce robust and brain-like computation and behavior^5–7^, while simultaneously producing a unique and different rendering of the planned movement with each iteration. This inherent variability mirrors the unpredictability and individuality seen in human artistic expression, where each stroke or gesture carries a distinct signature. This presents a positive feature when it comes to artistic production.

**Fig. 1:**
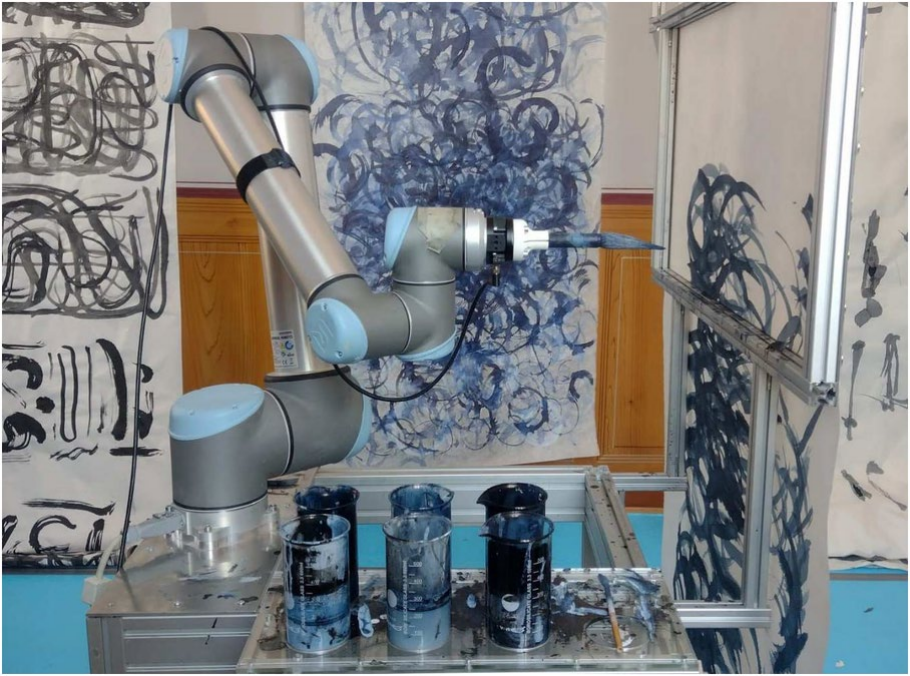
Physical setup comprising the magnetic painting frame and the 6-DOF robotic arm (UR10) with attached FT sensor and brush holder. Impression of the room installation Synaesthetic Strokes (2023) at the Collegium Helveticum. Photo by Marcus Nebe, ©VG Bild Kunst / Liat Grayver.

In this work, we implement a control system (see Fig. 1) for a robot arm to produce heterogeneous, and hence more natural, brushstrokes. The brushstrokes are produced using neural computational primitives implemented on the mixed-mode neuromorphic processor DYNAP-SE^8^, which emulates the dynamics of biological neurons and synapses in silico with biologically realistic dynamics. The silicon neurons on DYNAP-SE implement the Adaptive Exponential Integrate and Fire (AdExp I&F) neuron model, while the silicon synapses exhibit temporal dynamics, utilising first-order low-pass filters. Multiple neurons and synapses can be connected together through programmable routing tables to form Spiking Neural Networks (SNNs). In contrast to conventional artificial neural networks (ANNs), SNNs process information through their spike (or event) times and integrate temporal information present in their signals via synapses. To introduce variability in the generation of strokes and give a less artificial aspect, this project interfaced the physical painting machine to the DYNAP-SE, exploiting its analog nature. In pursuit of a human-like response from the system, coupled with the necessity for low-latency in robotics, the SNN implementation on neuromorphic electronic hardware represents a valuable approach towards achieving the desired objective.

Another drawback of existing painting machines is that the feedback used to optimize the overall painting plan is restricted to only visual inputs, whereas humans rely on both tactile (proprioceptive) and visual feedback at the level of individual brushstrokes. The integration of real-time visual feedback is often challenging due to the high complexity and occlusion caused by overlapping strokes in paintings. As an alternative to conventional cameras, event-based cameras (or Dynamic Vision Sensors – DVS) can significantly reduce the complexity of the visual scene^9^, as they only detect local changes in contrast on a per-pixel basis. As soon as the illumination contrast measured within a given pixel changes beyond a predefined threshold, a digital event is generated. Each event encodes the time of change detection, the spatial location (address), and the increase or decrease in contrast (polarity). The event-based representation of the scene automatically filters out the stationary part of the painting (i.e. the previous strokes) and only returns visual information about the most recent brushstroke being performed, thereby overcoming the limitation of traditional computer vision approaches to extract individual brushstrokes from complex scenes. Due to their sparse and asynchronous encoding with a stream of events, event-based cameras achieve temporal resolutions of microseconds and ultra-low power consumption^10^, making them ideal for robotic applications.

This work investigates the potential of using the change-driven scene representation provided by event cameras and the heterogeneity of spike-based neuromorphic processing can be exploited in an adaptive robotic system for the generation of brushstrokes that reproduce the heterogeneous quality of human painting. The robot observes the original brushstroke as it is being painted by an artist, extracting its inherent features, such as velocity, and produces a new unique brushstroke that is not simply a high-fidelity copy but preserves its artistic nature. The system incorporates both conventional digital robotic components as well as analog neuromorphic sensors and processors. To extend the feedback capabilities beyond those of previous painting machines, a force-torque sensor and an event-based camera are integrated into the controller. The proposed spiking controller ultimately computes a sequence of motor commands for the control of a 6-DoF robotic arm.

The project resulted in the artwork “Synaesthetic Strokes”, a collaborative effort between engineers and artists. It utilizes artificial systems inspired by the structure and functionality of biological neural networks to explore how the body and mind perceive not only the visual elements of the painting (the image) but also the intricate processes by which it was created - capturing both the form perceived by the eye and the energy felt within. This exploration aims to investigate the dynamic interplay between humanity and its media, forging a deeper understanding of the intricate connection between human expressiveness, creativity and technological innovation - from the traditional practices of manual craftsmanship to autonomous machine operation.

### Artistic motivation

The brushstroke, in all its diverse forms, serves as a singular communication tool encountered in paintings and drawings across different epochs. It is a key element in the development of our global and local cultural and perceptual aesthetic history. At the heart of this artistic practice is a profound exploration of the act of creating a painting itself, emphasizing the process of crafting a line, a brushstroke, rather than the final artwork.

Throughout the process of artistic exploration, passive materials respond to the active manipulation imposed upon them. Both the passive and active elements play equally significant roles in the creative process and the final outcome. Factors such as brush deformation, ink state, overlapping strokes, velocity, tilt, vectors, and pressure all contribute to the physical properties that are etched into the visual manifestation of the stroke itself. This complexity represents a difficult and open challenge for the design of artificial agents aimed to reproducing dynamic brushstrokes. An introduction to the historical background of robotic and AI-based painting is provided in the Supplementary Material section II. Unlike many similar projects done in the domain of robotic paintings - the goal of this project was not to develop a robotic system capable of perfectly replicating human brushstrokes with quantitative precision. Rather, by designing the project on physical knowledge, therefore the painting’s physical characteristics, from the realms of painting and calligraphy, this cross-disciplinary project aims to find a delicate equilibrium between material control, perception and the fluidity of bodily movements, allowing the unique characteristics of both gestural movements as well of paper and ink to guide the formation of each stroke. To distinguish from methods focused on concept perfection and execution in digital fabrication models, this project aims to highlight the imperfections of organic material behavior as they occur, informing and enhancing the artistic creative process. This adaptive behavior, which incorporates perceptual understanding, physical knowledge, and memory, highlights the subconscious, craft-oriented expertise that drives human creative improvisation and artistic expression. Given that creative practices, particularly painting, are inherently fluid and non-deterministic, traditional methods for achieving quantitatively accurate robotic replication were unsuitable. The long-term vision of creating such a system is to develop a “sensible” robotic painting system that can respond to the painterly process with each stroke, ultimately becoming a novel co-creator in an adaptive human-machine collaborative painterly process.

The experiments focus on a selected set of materials and scenarios to align with the goals of the research. The decision to limit the materials to ink, calligraphy brushes, and mulberry paper serves several purposes: The transparency of the 25g/m² half-treated mulberry paper allows real-time tracking of ink spread with neuromorphic camera positioned behind the paper. The calligraphy brush, with its versatile design, enables the creation of both thick and thin lines without the need to switch brushes, enhancing the fluidity of the process. Future iterations will focus on expanding artist-system interaction through real-time adjustment of brushstroke parameters and closed-loop feedback, incorporating usability studies and feedback from artists to strengthen the collaborative and intuitive aspects of the system. This aligns with the system’s design as a creative collaborator rather than a deterministic tool, emphasizing an ongoing dialogue where both human and machine adapt to each other’s actions and the evolving painting surface.

## Results

**Fig. 2:**
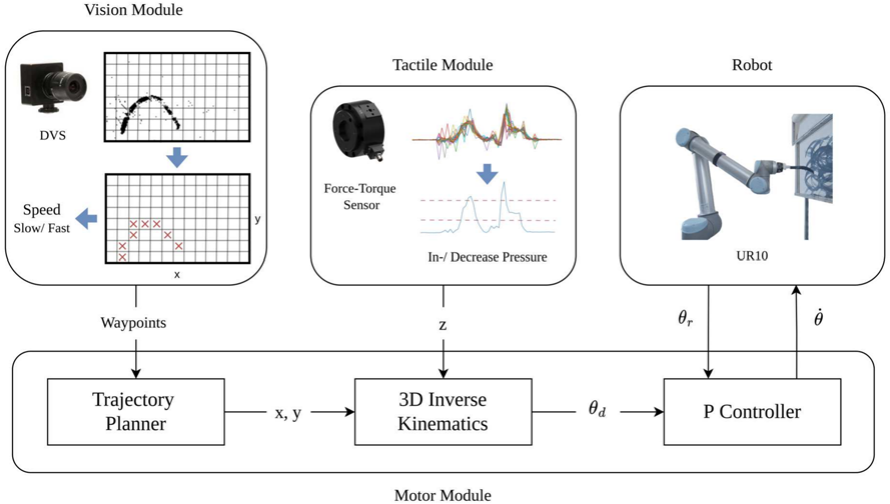
Overview of the proposed controller. The controller consists of a vision module, tactile module and motor module and communicates with a physical robotic system.

We propose a spiking closed-loop controller with multimodal sensory feedback for the generation of brushstrokes and demonstrate its implementation on the DYNAP-SE processor. The controller consists of three modules, as shown in Fig. 2: The vision module extracts the trajectory and speed of a detected brushstroke within the visual field of a DVS camera. Due to the high deformation of the painting brush, tactile feedback is essential for maintaining continuous contact between the brush and the paper. Therefore a tactile module processes sensory feedback from a Force-Torque (FT) sensor. Finally, the motor module is responsible for computing the required joint velocities depending on the robot’s current joint position. The architecture of the proposed spiking neural network is detailed in Section 3. For further details on the robotic setup please refer to Section III in Supplementary Material.

### Vision module

**Fig. 3:**
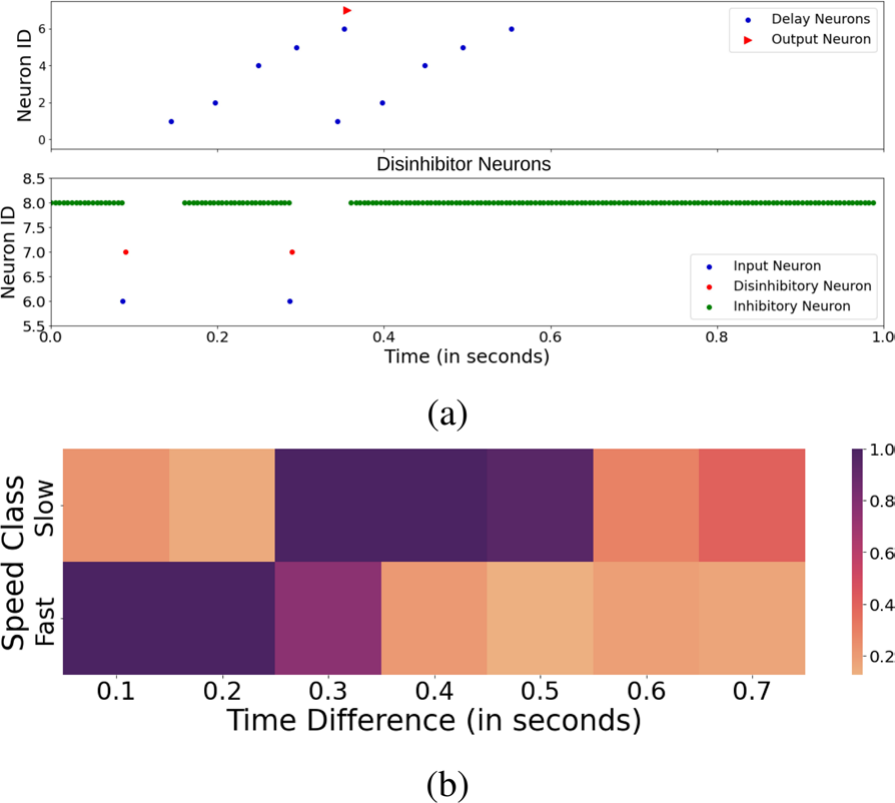
Experimental results of the Time Difference Encoding (TDE) unit. (**a**) The plot shows an example measurement of a TDE unit with sensitivity of 200 ms recorded on the DYNAP-SE 1 processor. The input neuron (bottom, blue dot) causes a suppression of the inhibitory neuron (bottom, green dots) and triggers a delay chain (top, blue dots). When the last neuron of the delay chain is active while the inhibitory neuron is suppressed, the output neuron (top, red triangle) is triggered as shown around time 0.35s. (**b**) Connectivity matrix between TDE units with sensitivities ranging from 200 ms to 700 ms and the two neurons representing the fast and slow speed classes after STDP learning. Each row corresponds to one TDE unit and each column to a different speed class. 1.0 indicates a maximum and 0.0 a minimum association between neurons.

The vision module leverages temporal information from the event-based camera to extract visual characteristics of the detected brushstroke. The speed at which a brushstroke is applied shapes the visual result of the generated artwork. Therefore, speed is crucial information for the painting process to be extracted from the vision sensor. The vision module extracts velocity information about the detected brushstroke from DVS events using a group of seven units employing time difference encoding (TDE) as described in section 3.2. Figure 3a illustrates an example measurement of a TDE unit implemented on the DYNAP-SE chip, in the form of a raster plot. The Figure shows only an explanatory example; with different input speeds and delays the raster plot would only differ in the lengths of the delay chain. The shown example employs a delay chain of length 5, resulting in a final sensitivity to time differences of 200 ms. The bottom plot depicts the suppression of the inhibitory neuron by the activity of the input and disinhibitory neurons. Simultaneously, the top plot depicts how the input neuron triggers a delay chain. When the final neuron within the chain, with the ID 6, fires while the inhibitory neuron remains suppressed, it activates the output neuron of the TDE unit. This can be seen in plot A around $t= 0.375$s.

For simplicity, the current implementation only assumes two classes of velocity - brushstrokes that are perceived as “slow” or “fast” by a human observer. To learn a mapping between the output neurons of the TDE units and the classification neurons representing the two-speed classes, a triplet STDP learning rule is applied. In this project, the implementation presented in^11^ is used. The DYNAP-SE chip is compatible with off-chip spike-timing-based learning rules, such as triplet STDP, which are well-suited to its hardware architecture, as the chip’s input and output signals are real-time streams of spikes. During the learning process, an array of spike times is randomly selected from a collected data set. The input neuron is excited at the designated spike times using a dedicated Field-Programmable Gate Array (FPGA) device as a spike generator. Simultaneously, either the slow or fast speed neuron is stimulated at a frequency of 200 Hz depending on the associated label of the collected spike times. For each class, 20 strokes, represented by their arrays of spike times, are utilized for learning. The connectivity matrix between the TDE output neurons and the speed neurons after learning is shown in Fig. 3b. The columns represent the output neurons of the TDE units that are sensitive to time differences ranging from 100 ms to 700 ms. The rows indicate the two-speed classes: “Slow” and “Fast”. The heatmap visualizes the synaptic connectivity, i.e. weights, between the input and output neurons after learning, with 1.0 indicating the maximum number of connections and 0.0 no connections. During learning, the triplet STDP learning rule leads to an increase of synapses between neurons that fire simultaneously and a decrease of connections between neurons that are uncorrelated. The matrix shows a stronger association of the “Slow” speed class with time differences of at least 300 ms, while the fast speed class is primarily centered around time differences of 100 ms and 200 ms. The learned weight matrix can then be used to classify the speed of strokes. Depending on the observed time differences of a brushstroke, either the “Slow” or “Fast” output neuron with the most incoming synapses will be activated. To allow a correct transition between individual strokes, the module must furthermore identify the end of a stroke. Such an event is characterized by the absence of a transition between visual states for at least one second. To determine the time delay between state transitions in the network, we propose an architecture based on delay chains which is detailed in the Supplementary Material along with the employed preprocessing pipeline in Section III-A.

#### Motor module

The motor module controls the robotic arm to follow the trajectory during the painting process. It utilizes a spiking inverse kinematics network followed by a P controller to determine a set of joint velocities that is continuously sent to the robot controller.

**Fig. 4:**
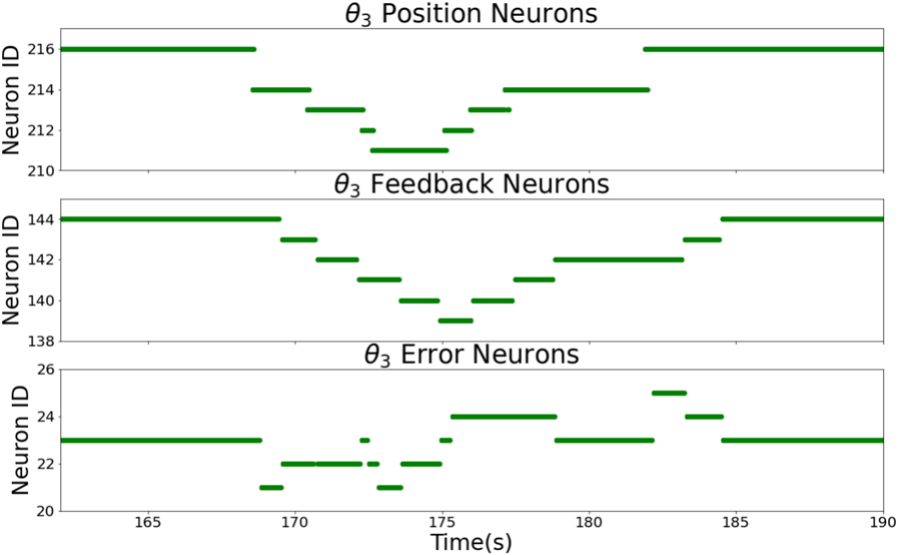
Raster plot visualizing the interaction between the $\theta _3$ error, $\theta _3$ feedback and $\theta _3$ position neuron populations within the spiking P controller. The controller computes the difference between the joint’s desired position (top) and its current position (middle) to determine the required motor command. This discrepancy is encoded by the error population (bottom). The neuron with ID 23 indicates that both positions are aligned.

The behaviour of the spiking P controller is visualized in more detail in Fig.  4. It illustrates the adaptive relationship between the (desired) position, feedback and error neuron populations, in the example of joint $\theta _3$ which corresponds to the elbow joint. The presented network employs one-hot encoding where each neuron represents a discrete value in the joint position space. Fig. 4 top and middle demonstrate how the activity of the feedback neurons (middle) - representing the actual joint position of the robot - follows the desired joint position. The error between the desired and actual position is captured by the error population as shown in the bottom plot. The neuron with ID 23 indicates that the error has converged to zero, implying the targeted alignment. An additional experiment demonstrating the variance introduced by the spiking controller is shown in the Supplementary Material in Section V.

#### Tactile module

**Fig. 5:**
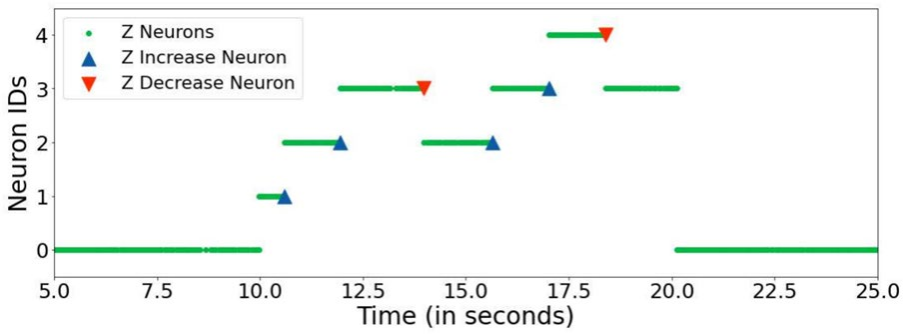
Raster plot illustrating the interaction between the z neurons and the z feedback neurons (z increase and z decrease) during a trial measurement on the DYNAP-SE 1 processor. The ID of the z neurons represents its associated z value in Cartesian space, with a higher ID indicating a larger z value. An activation of the z increase neurons (blue triangle) causes an increase of the z neurons (green dots) as seen around time 10.5, 12, 15.5 and 17 seconds. Conversely, an activation of the z decrease neurons (red triangle) causes a decrease of the z neurons (green dots) as shown around time 14 and 18 seconds. The z neuron with ID 0 indicates no contact with the paper or the end of a brushstroke (20 seconds). Z neurons with IDs between 1 and 4 (as shown between 10 and 20 seconds) encode a moving brushstroke and are sensitive to FT feedback.

Due to the high deformation of the painting brush, a continuous adaptation of the motor commands is required to maintain constant contact with the paper. To address this requirement, a Force-Torque (FT) sensor is integrated into the control pipeline which measures the force and torque applied along the sensor’s *x*, *y* and *z* axes and determines the current level of contact. In this setup we are utilizing the SensONE FT by Bota Systems (https://www.botasys.com/force-torque-sensors/sensone). Depending on the measured pressure levels, the desired *z*-axis position of the end effector is dynamically updated. The *z*-axis position directly corresponds to the brush’s distance to the paper surface. A population of Z neurons indicates the currently desired z axis position dependent on the input from the FT sensor and serves as input to the motor module. The network implementation of the tactile module is presented in detail in the Supplemental Material in Section IV. A raster plot visualizing the activity of the *z*, *z* increase and *z* decrease neuron populations is shown in Fig. 5. The activity was recorded while the robotic arm was painting on the physical canvas. The ID of the *z* neurons indicates the desired applied pressure, with a higher ID representing a larger pressure level. A single spike of the *z* increase neuron - signalling that the brush does not have sufficient contact with the canvas surface - causes an increase of the *z* neurons as shown around time 10.5, 12, 15.5 and 17 seconds. Conversely, an activation of the *z* decrease neurons causes a decrease of the *z* neurons. This can be seen in the plot around times 14 and 18 seconds.

A series of four exemplary brushstrokes illustrating the impact of the force feedback is presented in the Supplementary Material.

## Methods

This project explores the application of spiking neural networks and analog processing for physical painting machines, resulting in the artwork “Synaesthetic Strokes”. For further details on the robotic setup please refer to Section III in Supplementary Material.

### Physical setup

Figure 1 illustrates the painting setup established in this work. The setup comprises a UR10 robotic arm featuring six revolute joints, manufactured by Universal Robots (UR10 Universal Robots Datasheet). The robotic arm is fixed on a mobile table base which is extended by a magnetic frame. This frame comprises a specially designed aluminium structure covered by a metal layer and a second detachable plastic frame with embedded magnets that allow the fixation of a transparent sheet of paper between both frames. A paper roll can be attached on top of the frame, allowing for continuous painting and affording the human operator control to reposition the roll so that the robot has a blank (clean) section to paint on as needed. A 3D-printed brush holder securing two different sizes of calligraphic brushes and a force-torque (FT) sensor are mounted onto the robot’s end-effector. The FT sensor used in this project is the SensONE 6-axis sensor developed by Bota Systems (https://www.botasys.com/force-torque-sensors/sensone).

The setup additionally consists of a DVS camera placed on the opposite side of the magnetic frame and a lighting system that ensures a stable environment for visual data collection. The mobile base is further extended by a Plexiglas palette with six glass containers, holding sumi, blue and white ink, and two containers of water for each ink to dilute and create colour gradients. To explore different surface resistances and transparency effects, Chinese Xuan rice paper and Mulberry paper are employed. The neuromorphic DYNAP-SE 1 processor is employed for sensory processing and for the generation of motor commands to control the robotic arm. The processor comprises four cores with a total of 1024 Integrate and Fire (IF) neurons. Individual neuron units can be connected via excitatory or inhibitory synaptic circuits and each core is configurable via a separate set of shared neural and synaptic parameters. A computer-in-the-loop is responsible for facilitating communication between the processor, sensors and the robotic system, as well as for converting data between the digital interface of the physical robot and the force-torque sensor and event-based data.

### Neural architecture

#### Vision module

The vision module processes visual events from a DVS camera to extract the trajectory and speed of a detected brushstroke and to determine whether the painting movement has ended. The DVS events are sent to the DYNAP-SE processor through a computer-in-the-loop and subsampled onto a smaller visual field to fit the activity to the number of neurons and synapses available on the chip. To determine the speed of a detected brushstroke and to signal the controller whether the stroke has ended, it is necessary to understand the precise timing when a brushstroke has transitioned into a new visual state, i.e. to a new coordinate point in the subsampled space. A dedicated population of neurons called State Transition Neurons is set up to indicate such a transition, where each neuron represents one coordinate point p(x,y) in the subsampled visual field. The subsampling and state transition mechanisms are described in detail in the supplemental material.

##### Time difference encoding units for speed detection

**Fig. 6:**
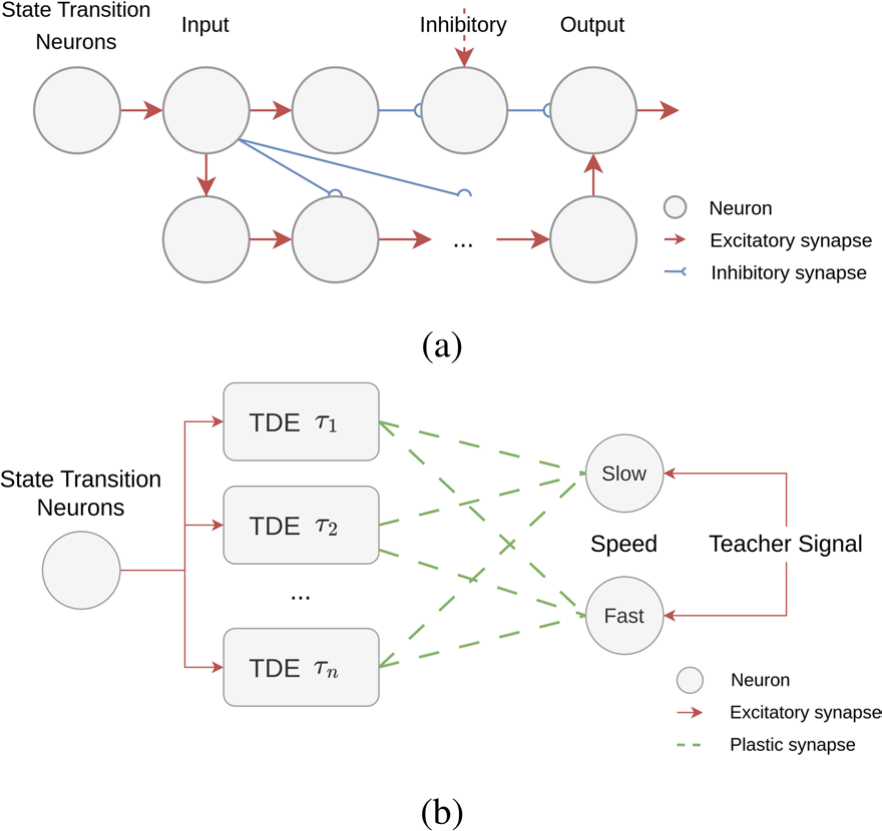
Network architecture for speed detection. (**a**) Network architecture inspired by the Time Difference Encoding (TDE) unit^13–15^. The input neuron disinhibits the output neuron and triggers a delay chain. The output neuron receives excitatory input from the last neuron of the chain. It is triggered when the input neuron is consecutively activated and the interspike interval aligns with the time it takes a spike to propagate along the delay chain. (**b**) The STDP pipeline learns to associate the output neurons of different TDE units with the speed classes Slow and Fast. Each TDE unit is sensitive to different time differences. The plastic synapses are strengthened whenever an output and speed neuron are active at the same time.

The speed of a brushstroke is inversely proportional to the motion time of the brush between visual locations and can be determined with time difference encoding (TDE)^12,13^ neurons. These neurons measure the time to travel of a visual stimulus between two visual locations. TDE neurons can be realised with specialised circuits, such as Milde et al.’s circuit^14^ based on the Differential Pair Integrator (DPI)^15^ which encodes time to travel in the firing rate of output spike bursts. Due to the absence of such a circuit on the DYNAP-SE processor, a delay chain is implemented on the processor to encode time to travel for speed detection. The precise timing of transitions in the visual scene can be extracted by the state transition network as described earlier. Figure 6a shows the proposed TDE unit based on a delay chain. The unit’s output neuron is constantly suppressed by neuron *Inh*. As soon as the input neuron is activated, it disinhibits the output neuron via an interneuron. At the same time, it re-triggers a chain of excitatory neurons. The last neuron of this delay chain has an excitatory connection to the output neuron. It can activate the output neuron only if the neuron is simultaneously disinhibited by the input neuron. This condition is met when the time difference between two consecutive input spikes aligns with the time it takes a spike to propagate along the delay chain. Consequently, the activation of the output neuron signals a set time difference between individual spikes. Using delay chains of different lengths, the TDE units detect different time differences, and, therefore, different speeds.

To extract speed information from the visual sensor using this computational unit, multiple TDE units each sensitive to different time delays in the range of [100$\div$700]ms are employed. The state transition neurons are connected to each TDE unit via all-to-one synaptic connections, independent of their spatial location. The incorporation of speed information is crucial to represent the robot’s movements in a human-like stroke. To simplify this representation, a binary categorization of speed, perceived as either “fast” or “slow” by the human painter, is adopted. When a brushstroke is initiated, it triggers a sequence of time differences with varying duration. Consequently, the challenge at hand involves establishing a meaningful correlation between these temporal differences and speed detection. In response to this challenge, a binary classification system featuring two neurons, each representing the fast and slow speed classes, has been implemented.

As a learning paradigm, triplet Spike-Timing-Dependent Plasticity (STDP)^16^ is employed to determine the connectivity between the speed neurons and the output neurons of the TDE units as visualized in Fig. 6b. STDP is a biologically plausible form of Hebbian learning which modifies the synaptic strength between two neurons based on the relative timing of pre and post-synaptic spikes. STDP is supported by the DYNAP-SE 1 processor via a computer-in-the-loop setup that changes the chip’s parameters according to the learning rule implemented in software on the computer. The triplet STDP rule has previously been utilized to successfully learn an inverse kinematics mapping on the DYNAP-SE chip^11^. While regular STDP requires the presynaptic neuron to repeatedly fire first, triplet STDP incorporates the timing of a second spike at the postsynaptic neuron, focusing the update rule on simultaneous activation. For the learning process, a data set of human-generated brushstrokes at visually perceived “slow” and “fast” strokes is recorded. The time delays of the individual brushstrokes serve as input to the TDE units. Concurrently, the speed neuron corresponding to the stroke’s appropriate label is stimulated. The connectivity is therefore strengthened when the simultaneous activity of the speed neuron and the output neuron of the TDE unit sensitive to that specific time difference occurs. This synaptic reinforcement enables the network to associate specific time differences with the corresponding speed classification, effectively learning to decode speed information based on the temporal dynamics of the sensor data. To learn the time differences associated with strokes that are perceived as either “slow” or “fast”, a total of seven TDE units are implemented on the DYNAP-SE processor. Each unit consists of an increasing number of delay neurons with the same time constants. The delay of the unit is dependent on the length of this delay chain. In this work, we investigate units with time differences ranging from 100 ms to 700 ms at a step size of 100 ms. Understanding and associating event delays in both slow and fast motion of the painted brushstroke allows us to ensure that the painting speed remains within the human range, thereby avoiding damage to the canvas.

#### Motor module

The motor module is responsible for the joint velocity control of the robotic arm and dynamically computes the required joint velocity commands depending on the robot’s current and desired position. It consists of a trajectory planner, a 3D inverse kinematics network and a spiking P controller. The trajectory planner calculates an optimal path between the robot’s current position in Cartesian space and the target waypoint the robot has to reach. As output, it provides the next (*x*, *y*) coordinate on this trajectory. The 3D inverse kinematics network converts a 3D Cartesian coordinate into the robot’s joint space. It receives as input the previously determined (*x*, *y*) coordinate as well as the desired *z*-axis value which is computed by the tactile module. The P controller finally calculates the difference between the robot’s actual and desired position for all five joints. Based on the computed position errors, a set of joint velocities to be sent to the robotic arm is determined.

Previous works have demonstrated how classical P(ID) controllers can be implemented with spiking neural networks and verified their applicability for robotic control. Zhao et al. demonstrate how a PID controller can be implemented with relational networks on the DYNAP-SE processor to control a robotic arm.^11^. One essential component of their proposed controller is a relational network which links two 1D input populations to a 1D output population via a 2D hidden layer of neurons. This network architecture enables various operations between input and output populations, such as computing the error between desired and current joint positions. Inspired by this approach, this work employs the concept of relational networks to implement the inverse kinematics and P controller network of the motor module.

To find an optimal trajectory in Cartesian space, the proposed controller uses a wavefront-based path planner^17^, an algorithm based on the concept that a wave uniformly propagates in its environment. Consequently, it assumes that the first wave to hit any location in space has travelled along an optimal trajectory between its point of origin and this location. The parallel nature of the algorithm makes it particularly suitable for neuromorphic processors which are inherently parallel. The wavefront path planner has previously been implemented successfully on neuromorphic hardware, such as IBM’s TrueNorth processor^18^.

##### Trajectory planning network

The main component of the trajectory planning network is a population of state neurons in which each neuron represents one coordinate point *p*(*x*, *y*) on the canvas. The state population is implemented as a neural state machine as introduced by Liang et al.^19^, a spike-based implementation of a finite state machine representing the currently desired 2D position. As soon as a new waypoint $p'(x',y')$ is received from the goal population, the network updates the robot’s state to the next position on the optimal trajectory to reach the waypoint. Each neuron in the goal population represents a coordinate point.

**Fig. 7:**
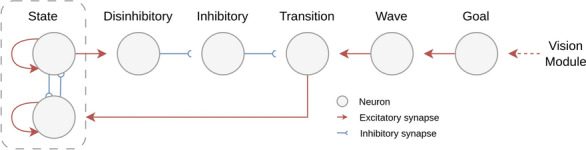
Pipeline of the trajectory planning network. Each neuron in the state, disinhibitory, inhibitory, transition and wave populations represents a 2D coordinate point p(x,y) on the canvas. The state neurons disinhibit all transition neurons that corresponding to directly adjacent points, therefore all the neighbouring neurons. If a transition neuron is activated by the wave population, it triggers an update of the state population to a new coordinate point p’(x’,y’) that has to be reached by the robot’s end-effector.

The wavefront algorithm is implemented as a spiking neural network on the DYNAP-SE chip using the network architecture shown in Fig. 7. It consists of a total of five neural populations, namely: state, disinhibitory, inhibitory, transition and wave population; in which every population consists of neurons representing the entire coordinate space. Every state neuron has excitatory connections to all its corresponding neighbours in the disinhibitory population. The disinhibitory population has to inhibit one-to-one connections to the inhibitory neurons which in turn have to inhibit one-to-one connections to the transition population. The inhibitory neurons additionally receive continuous external stimulation from the FPGA and are constantly active if not inhibited by the disinhibitory population. As the robot’s current state is constantly active, its neighbouring neurons in the transition population are the only transition neurons that remain disinhibited.

The key element of the wavefront algorithm is the wave population, in which every neuron has excitatory connections to all its neighbours. The activation of a single wave neuron consequently triggers a propagating wavefront through the neuron population. To ensure that direct neighbours are preferred over diagonal neighbours when looking for an optimal trajectory, direct neighbours are connected via fast excitatory synapses, whereas diagonal neighbours have slow excitatory connections. As soon as a new waypoint is requested, the goal population initiates a propagating wave front in the wave population. Due to the inhibition of the transition layer, the first transition neuron that gets triggered by a wave neuron must be a neighbour of the currently active state neuron, representing a directly adjacent point on the canvas. Because of the uniform propagation of the wavefront, this transition neuron represents the closest neighbour to the wave’s origin and hence lies on the optimal trajectory to the new waypoint. The activated transition neuron in turn excites its corresponding neuron in the state population and inhibits the currently active state neuron. This leads to an update of the robot’s desired coordinate point.

##### 3D inverse kinematics network

The current state of the trajectory planning network defines the desired 2D position of the robot’s end effector. Additionally, the desired *z* position is determined by the controller’s tactile module. The 3D inverse kinematics network is responsible for transforming the desired 3D position in Cartesian space into a corresponding set of joint positions. In the case of the UR10 robot (see Fig. 1), an analytical solution for its inverse kinematics exists. To ensure that only a single joint configuration exists for a desired pose, the following restrictions are introduced:

1. The shoulder joint has to be turned to the left to avoid collision with the ink containers
2. The elbow has to point upwards to avoid collision with the base
3. The wrist 1 joint points downwards

With the help of the analytical solution, the required joint configurations for every possible pose within the workspace can be therefore calculated.

**Fig. 8:**
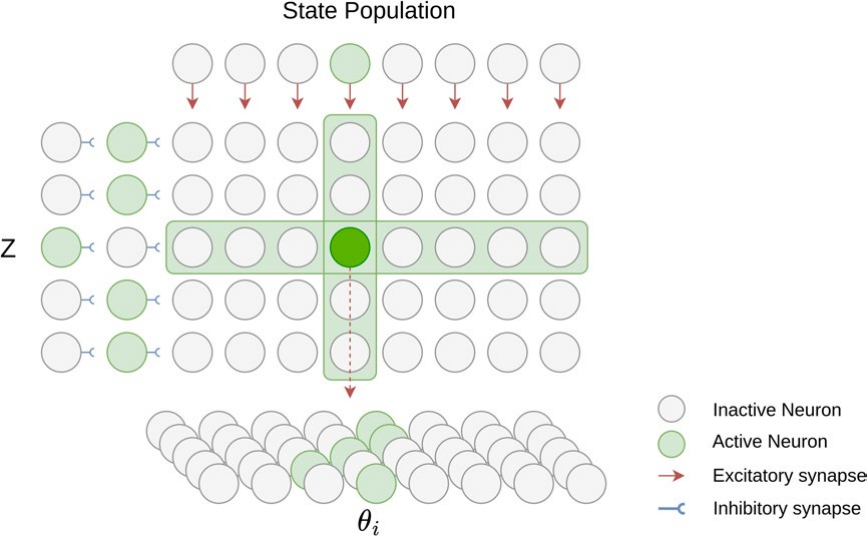
Architecture of the 3D inverse kinematics network. The network computes the joint positions necessary for the robot’s end-effector to reach a target 3D coordinate point p(x, y, z) in Cartesian space. A hidden population maps each possible 3D point to a unique set of joint positions. Every column of the hidden population receives excitatory input from a single neuron of the state population, representing a 2D point p(x,y). Each z neuron disinhibits one row of the hidden population. The hidden neuron that is disinhibited by a z neuron and excited by a state neuron at the same time stimulates a joint position neuron for all five joints, encoding the joint positions that are required to reach the 3D point p(x,y,z).

A population of joint position neurons is defined for every joint of the robot, where each neuron represents a discrete position. These populations consequently represent a discretization of the supported range of joint positions. To map a 3D coordinate to its corresponding joint configuration, the relational network shown in Fig. 8 is used. The 1-dimensional state and *z*-neuron populations are connected to a 2-dimensional hidden population. Every neuron of the state population has an excitatory connection to one column of the hidden population, while every *z*-neuron disinhibits one hidden row. Due to this setup, every neuron of the hidden population can only be stimulated if, and only if, both a state neuron and a *z*-neuron are active at the same time. Every neuron of the hidden population in turn activates one neuron in each of the robot’s $\theta _i$ joint position populations (in this case the correct connection is computed algorithmically, as described above, but could be learnt as in^11^), indicating the configuration required to reach the desired 3D pose.

##### Spiking P controller

A spiking P controller is employed to achieve the final step of the motor controller, determining the next set of joint velocity commands to be sent to the robotic arm. The P controller adjusts the system’s control output proportionally to the error between its desired and current output but does not take into account the system’s past or predicted future behaviour. One P controller network per joint is implemented, following the same relational network structure as the Inverse Kinematics Network in Fig. 8. The P controller network receives as input the desired joint positions $\theta _{desired}$ and the robot’s current joint positions $\theta _{robot}^{\circ }$ The output of the inverse kinematics network determines the desired joint positions. Both populations are implemented following a one-hot encoding scheme where every neuron represents one possible joint position.

As the robotic arm does not support direct incoming or outgoing spikes, the activity of the $\theta _{robot}$ populations is updated via a computer-in-the-loop. The computer receives the robot’s current joint state, determines the position neuron representing the closest configuration and finally stimulates the corresponding $\theta _{robot}$ feedback neuron on the chip. Each $\theta _{robot}$ neuron excites one row of the P controller’s hidden population, while every $\theta _{desired}$ neuron disinhibits one column. The hidden neuron that is excited and disinhibited at the same time stimulates the error neuron representing the difference between the current and desired joint position. Due to the limited number of neurons on the DYNAP-SE processor, the supported error values are limited to the range $[-0.3, 0.3]$ radians with step size 0.1. If the calculated difference is larger, the hidden neuron maps to the error neuron with the closest value. The resulting position errors are read out by a computer-in-the-loop and linearly mapped onto a corresponding joint velocity value. These velocity commands are finally sent as a motor command to the robot.

## Video

A video of the entire project “Synaesthetic Strokes” and the exhibition can be found here https://www.youtube.com/watch?v=G9jdB_xVWqE&ab_channel=liatgrayver.

## Discussion

In this work, a spike-based controller with multimodal sensory input for the generation of physical brushstrokes with a robotic arm is presented. The proposed controller employs spiking neural networks on a neuromorphic platform that processes input from an event-based camera and a force-torque sensor and computes joint velocity commands for the control of a 6DOF arm. The project demonstrates how the inherent temporality of spiking neural networks can be utilized to extract temporal information about its sensory input. Unlike artificial neural networks, spiking neurons are not only defined by their activation but also their precise spike times, allowing a direct representation of time within the network. This work implements two network architectures that encode time delays to determine the speed and the end of a detected brushstroke respectively. The classification of slow and fast motion revealed that human brushstrokes perceived as “slow” were achieved by time delays of 0.3-0.5 s between visual states and brushstrokes perceived as “fast” consisted of time delays below 0.3 s (see Fig. 3b). The results show that slow human brushstrokes rarely ever exceed the time difference of 0.5 s, revealing the speed range of human painting motion. Another major difference between the employed analog neuromorphic processor and conventional processing units is its non-deterministic nature. This causes slight variations in the precise spike times of the neurons and consequently in the resulting behaviour of the controller. When comparing the trajectory of the robot’s end-effector across multiple trial runs (Fig. S8 in the Supplementary Material), the generated trajectories are never identical (Fig. S7 in the Supplementary Material) despite the controller receiving the same path. In the use case of painting, this non-determinism is a desired feature, resulting in unique yet analogous replications of a brushstroke instead of mere copies.

The proposed control architecture exhibits two major limitations, namely its latency and its discretized representation of the workspace. The latency limitation stems from the fact that the interfaces of the force-torque sensor, the event-based camera and the robotic controller do not operate in a spike-based manner and therefore require a spike conversion prior to their processing on the neuromorphic processor. During the conversion, sensory inputs and outputs are aggregated over a set time window and subsequently encoded or decoded into a spike train.

In the presented experiments, input event streams from the DVS camera and torque measurements from the FT sensor are aggregated for 33ms and 330ms respectively before converting and forwarding them to the neuromorphic processor, while the joint velocity commands are updated and sent to the robotic arm at a frequency of 20 Hz. The large aggregation window of the FT sensor is due to the requirement of a minimum of 33 data points to low-pass filter the raw sensor data before conversion. In addition to these predefined aggregation windows, the USB interfaces between the computer-in-the-loop and the sensors and controller introduce an additional delay.

This undesirable latency in the processing pipeline undermines the advantages associated with the ultra-high temporal resolution capabilities of neuromorphic processors and sensors. However, most of the delay can be removed by replacing the conversion between digital and spike-based representations on a computer-in-the-loop with direct AER interfaces to the neuromorphic processor in a fully end-to-end spiking pipeline. In a previous work, a custom FPGA board has been presented to directly interface the DVS camera with the Dynap-SE processor^20^, restricting the latency to only the propagation speed of spikes on the processor. Zhao et al. investigate the trade-off between latency, accuracy and power consumption of a similar inverse kinematic network on the same neuromorphic processor in their work^11^. Their network consists of a second hidden layer and learns the connections instead of determining them algorithmically. They achieve 33.96 ms on-chip network latency and 26.92 $\mu W$ on-chip power consumption with a network of 184 neurons of which 16% are active. In comparison, our inverse kinematics network consists of 305 neurons of which 2% are active, however with a higher firing rate. Additionally, the proposed network was not tuned for minimizing latency and while the latency could be further improved, it is sufficient for many robotic manipulation tasks.

Furthermore, the presented network employs a one-hot encoding strategy to represent the Cartesian space of the painting canvas and the robot’s joint space through neuron populations. This approach entails the discretization of the Cartesian and joint spaces with each state being uniquely represented by an individual neuron. To investigate the impact of the discretization on the performance of the spiking P controller, the controller was tasked to follow a discrete sinusoidal wave. When comparing the joint’s actual position with the desired position of the sinusoidal wave, the discretization leads to a steady-state error of approximately 0.3 radians. Consequently, the discretization results in deviations from the intended brush trajectory during the painting process. Although we describe this differentiation as a limitation, a certain degree of discretization is desired to obtain more heterogeneous human-like brushstrokes.

The access to ultra-low latency feedback from the visual scene could additionally help compensate for unreliable sensor data. The current system is primarily reliant on feedback from an FT sensor to regulate the desired distance towards the canvas. However, this approach has shown to be insufficient in situations with minimal contact where the sensor receives near-zero input. The integration of multi-sensory feedback from both an FT and vision sensor could overcome this limitation. Discretization errors could furthermore be mitigated by replacing the one-hot encoding of the Cartesian and joint space with population encoding. Instead of monitoring the presence or absence of firing in individual neurons, the firing rate of a population of spiking neurons would help encode the desired coordinates and joint positions, leading to a more nuanced representation of the workspace.

### Artistic vision and final exhibition

The proposed controller was used for the realisation of the artwork “Synaesthetic Strokes” which explores the integration of traditional craftsmanship principles to create robotic-based paintings. One essential component of the established painting studio for this project involves attaching the paper roll on top of the aluminium frame which acts as a canvas. This setup allows the dynamic growth of the painting and provides the human operator with the ability to subjectively determine when the paper is sufficiently saturated or æsthetically pleasing. At this point, the artist can pause the painting process, scroll down to a clean section of the paper and allow the machine to continue generating new strokes. This interactive setup empowers the artist to actively engage with the machine in shaping the artistic output, highlighting the collaborative nature of the artwork. It combines the capabilities of the machine with the subjective decisions and artistic sensibilities of the human operator, resulting in a unique and evolving artistic creation.

Upon painting approximately 5-7 metres of paper, the painting was cut from the long scroll and suspended from the ceiling to the floor of the historical building at the ETH Observatory, where the exhibition was held (see Fig. 1). This arrangement gradually filled the entire room with a combination of mechanical and human-made strokes, immersing visitors in a sea of brushstrokes and challenging them to discern between those created by the machine and those by a human. The overlapping of strokes, whether wet on dry or wet on wet, results in the formation of intricate and complex compositions, 1 m wide and up to 7 metres in length. This composition emerges from a fusion of algorithmic procedures, the intricate behaviours inherent in the materials and the machine’s perceptive understanding of the stroke. The outcome is an organic evolution of strokes, embodying the dynamic interplay between human creativity and decision-making in conjunction with machine processes. The resulting performative artwork embodies a dynamic and collaborative exploration between humans and artificial systems, deconstructing the essence of a brushstroke and provoking enquiries into its nature. It prompts reflection on the perception and replication of brushstrokes by artificial entities, as well as the profound significance of the technical complexities inherent in this seemingly simple task. These challenges bear relevance not only in the context of rapid advancements in autonomous machines but also in understanding the capabilities and boundaries of human agency. The network size of both the inverse kinematics network and the wavefront path planner scale linearly with the size of the workspace, depending on the chosen discretization step. The wavefront algorithm triggers a new spike wave for each trajectory point of the brushstroke. The computation time and cost of the algorithm are therefore also dependent on the length *n* of the brushstroke. The computation time has an upper bound of *O*(*mnl*), where l is the latency of a single network transition and m is *max*(*width*, *length*) of the canvas. Due to the inherently parallel nature of the neuromorphic processor, only the maximum width or length has to be considered. While our current implementation leverages the DYNAP-SE neuromorphic chip, the proposed control system is adaptable to a range of neuromorphic platforms. Depending on the number of degrees of freedom and task complexity, the spiking architecture can be distributed across multiple chips or scaled on higher-capacity hardware such as Loihi or SpiNNaker. This adaptability comes with a trade-off: mixed-signal neuromorphic chips like DYNAP-SE offer inherent noise and mismatch that contribute to human-like variability, a feature that may be lost on purely digital platforms.

## Conclusion

The proposed controller represents an initial step towards a fully spiking robotic system with the ability to seamlessly incorporate event-based sensory feedback, providing ultra-low latency responsiveness.

A biologically-inspired event-based camera captures the 2D trajectory and speed of a detected brushstroke and provides visual input to the system, therefore creating a fully bio-inspired system. Additionally, a force-torque sensor is attached to the robot’s end-effector to capture the pressure applied to the paper during the painting process. Based on this tactile feedback, the controller dynamically adjusts the desired distance between the brush and the canvas, ensuring constant contact with the paper. Finally, the motor module computes the joint velocities required to replicate the desired brush trajectory. It consists of a trajectory planner - ensuring a smooth transition between individual brushstrokes -, a 3D inverse kinematics network, and a spiking P controller which employs joint velocity control based on the robot’s current joint position. Beyond its utility in robot-assisted painting, the presented network applies to any robotic task requiring real-time adaptive control.

The primary artistic objective of this project was the development of an adaptive artificial behaviour system that incorporates principles of traditional craftsmanship to create robotic-based paintings. These paintings serve as a foundation for training the machine to achieve complex and nuanced outcomes and reactions throughout the painterly process. The long-term goal of this project is the creation of a brushstroke depository, or library, of “gestural” robotic movements (tactile-physical) and presets of rules and “conditions” for their execution (perceptual-cultural), an accessible platform that will be built later for broader use in robotic painting (both hardware and software). As the library will compartmentalize not only individual brushstrokes as gestures but also visual information, it will be possible to integrate the repository into the visual feedback mechanism of another robotic painting interface. As an example of the many important implications this holds for the future of robotic painting, this will afford users the possibility to decide on the level of human control or autonomous robotic painting creation, as it will be possible to set rules for the use of individually defined variants of stroke characteristics. This exploration aimed to redefine the traditional art form of painting in the context of our technology-driven era while examining the influence of robots as interactive painterly tools on creativity, authorship and agency in artificial systems. Moreover, the proposed neuromorphic controller can be readily adapted to other robotic manipulation tasks that rely on Cartesian position control, particularly those where precise, real-time operation is not critical, such as assistive robotics, artistic applications, and exploration tasks in unstructured environments. In conclusion, in a future implementation, the system could also benefit from reinforcement learning (RL) approaches, as demonstrated by Zendrikov et al.^21^, to further enhance performance and adaptability.

## Supplementary Information

Supplementary Information.

## Data Availability

The data used in the study is available on request from the corresponding author.

## References

[1] Ramesh, A., Pavlov, M., Goh, G. et al. *Zero-Shot Text-to-Image Generation*, arXiv:2102.12092 [cs], (2021). (Accessed 21 July 2023).

[2] Gülzow, J. M., Paetzold, P. & Deussen, O. Recent developments regarding painting robots for research in automatic painting, artificial creativity, and machine learning. *Appl. Sci.* **10**(10), 3396. 10.3390/app10103396 (2020).

[3] Schaldenbrand, P., McCann, J. & Oh, J. *FRIDA: A Collaborative Robot Painter with a Differentiable, Real2Sim2Real Planning Environment* arXiv:2210.00664 [cs], (2022). (Accessed 21 July 2023).

[4] Carter, R. & Carter, N. Dark factory paintings. http://www.%20robandnick.com/dark-factory-portraits (2017).

[5] Liu, S.-C. & Delbruck, T. Neuromorphic sensory systems. *Curr. Opin. Neurobiol.* **20**(3), 288–295. 10.1016/j.conb.2010.03.007 (2010). 20493680 10.1016/j.conb.2010.03.007

[6] Chicca, E., Stefanini, F., Bartolozzi, C. & Indiveri, G. Neuromorphic electronic circuits for building autonomous cognitive systems. *Proc. IEEE* **102**(9), 1367–1388. 10.1109/JPROC.2014.2313954 (2014).

[7] Zendrikov, D., Solinas, S. & Indiveri, G. Brain-inspired methods for achieving robust computation in heterogeneous mixed-signal neuromorphic processing systems. *Neuromorphic Comput. Eng.* **3**(3), 034002. 10.1088/2634-4386/ace64c (2023).

[8] Moradi, S., Qiao, N., Stefanini, F. & Indiveri, G. A scalable multicore architecture with heterogeneous memory structures for dynamic neuromorphic asynchronous processors (DYNAPs). *IEEE Trans. Biomed. Circuits Syst.* **12**(1), 106–122. 10.1109/TBCAS.2017.2759700 (2018). 29377800 10.1109/TBCAS.2017.2759700

[9] Lichtsteiner, P., Posch, C. & Delbruck, T. A 128x128 120 dB 30 mW asynchronous vision sensor that responds to relative intensity change. In *2006 IEEE ISSCC Digest of Technical Papers* 508–509 (IEEE, 2006).

[10] Gava, L., Monforte, M., Bartolozzi, C. & Glover, A. How late is too late? a preliminary event-based latency evaluation. In *2022 8th International Conference on Event-Based Control, Communication, and Signal Processing (EBCCSP)*, 1–4 (IEEE, 2022).

[11] Zhao, J., Monforte, M., Indiveri, G., Bartolozzi, C. & Donati, E. Learning inverse kinematics using neural computational primitives on neuromorphic hardware. *npj Robot.*10.1038/s44182-023-00001-w (2023).

[12] Kramer, J., Sarpeshkar, R. & Koch, C. Pulse-based analog VLSI velocity sensors. *IEEE Trans. Circuits Syst. II* **44**(2), 86–101 (1997).

[13] D’Angelo, G. et al. Event-based eccentric motion detection exploiting time difference encoding. *Front. Neurosci.* **14**, 451 (2020). 32457575 10.3389/fnins.2020.00451 PMC7227134

[14] Milde, M. B., Bertrand, O. J. N., Ramachandran, H., Egelhaaf, M. & Chicca, E. Spiking elementary motion detector in neuromorphic systems. *Neural Comput.* **30**(9), 2384–2417. 10.1162/neco_a_01112 (2018). 30021082 10.1162/neco_a_01112

[15] Bartolozzi, C. & Indiveri, G. Synaptic dynamics in analog vlsi. *Neural Comput.* **19**(10), 2581–2603 (2007). 17716003 10.1162/neco.2007.19.10.2581

[16] Gjorgjieva, J., Clopath, C., Audet, J. & Pfister, J.-P. A triplet spike-timing-dependent plasticity model generalizes the Bienenstock-Cooper-Munro rule to higher-order spatiotemporal correlations. *Proc. Natl. Acad. Sci.* **108**(48), 19383–19388 (2011). 22080608 10.1073/pnas.1105933108 PMC3228426

[17] Ponulak, F. & Hopfield, J. Rapid, parallel path planning by propagating wavefronts of spiking neural activity. *Front. Comput. Neurosci.*10.3389/fncom.2013.00098 (2013). 23882213 10.3389/fncom.2013.00098 PMC3714542

[18] Fischl, K. D., Fair, K., Tsai, W.-Y., Sampson, J. & Andreou, A. Path planning on the TrueNorth neurosynaptic system. In *2017 IEEE International Symposium on Circuits and Systems (ISCAS)*, ISSN: 2379-447X, 1–4 10.1109/ISCAS.2017.8050932 (2017).

[19] Liang, D. & Indiveri, G. Robust state-dependent computation in neuromorphic electronic systems. In *2017 IEEE Biomedical Circuits and Systems Conference (BioCAS)*, 1–4 10.1109/BIOCAS.2017.8325075 (2017).

[20] Risi, N., Aimar, A., Donati, E., Solinas, S. & Indiveri, G. A spike-based neuromorphic architecture of stereo vision. *Front. Neurorobot.*10.3389/fnbot.2020.568283 (2020). 33304262 10.3389/fnbot.2020.568283 PMC7693562

[21] Zendrikov, D. Principles of robust neural computation through the lens of analog neuromorphic hardware. Ph.D. dissertation, (University of Zurich, 2024).

