# Supplementary material for: A neuromorphic electronic artist for robotic painting
Source: Sci Rep. 2025 Jun 4;15:19561. doi: 10.1038/s41598-025-92081-x (PMC12137699; doi:10.1038/s41598-025-92081-x)
Supplement: Supplementary file 1 — Supplementary Information. [file 41598_2025_92081_MOESM1_ESM.pdf]

# A neuromorphic electronic artist for robotic painting - Supplementary Material

Lioba Schürmann[1*], Giulia D'Angelo[2,3*], Liat Grayver[1,4], Chiara Bartolozzi[2], Giacomo Indiveri[1]
[1]Institute of Neuroinformatics, University of Zurich and ETH, Zurich, Switzerland
[2] Event-Driven Perception for Robotics, Italian Institute of Technology, Genoa, Italy
[3] Department of Cybernetics, Faculty of Electrical Engineering, Czech Technical University, Prague, Czech Republic
[4] Collegium Helveticum, Zurich, Switzerland

## I. Network Parameters

Figure 1 shows an extract of the employed synaptic parameters in the proposed spiking neural network, focusing on the P controller, inverse kinematics and TDE network. The Dynap-SE1 neuromorphic processor supports four types of synaptic connections, namely fast excitatory (AMPA inspired), slow excitatory (NMDA inspired), fast inhibitory (GABA A inspired) and slow inhibitory (GABA B inspired) synapses. The connection strength is dependent on the synapse's time constant tau, spiking threshold and weight. These parameters can be set indirectly by setting their coarse and fine values as shown in the table for the example networks. The coarse values are in the range [0,7], while the fine values have a range of [0,255]. A detailed overview of the processor's circuits is provided in [1].

## II. Historical Background and Artistic Context

In 1967, Marshall McLuhan proposed that "All media are extensions of some human faculty — psychic or physical" and, further, that "electric circuitry [is] an extension of the central nervous system" [2]. The broader field of arts, particularly within those areas with a focus on craft-based practices, is currently witnessing an unprecedented set of challenges and opportunities presented by automated technologies. Artists should investigate these emerging creation and fabrication methodologies to be able to collaborate and work alongside different degrees of Artificial Intelligence (AI).

Embracing robotic systems operated by AI in the artistic process presents an opportunity to transcend the limitations of the purely digital medium of image production and delve into new realms of artistic exploration. By incorporating artistic concepts as subjects of investigation and potential re-evaluation in the context of AI, we can push the boundaries of our understanding and challenge traditional notions of creativity. By creating an interactive and adaptive platform (co-painting with a machine) and working with implicitly complex materials and tools (paint and brushes), this project wishes to add complexity and surprise into the outcome of the work. Through interactive human-robotic painting, this approach goes beyond conventional approaches to AI in art, that increase, expand and recreate stochastic forms of existing cultural-æsthetic tendencies and artefacts, by the use of algorithmic procedures to produce geometrical forms and patterns that merge deterministic operations, random/infinite concepts and forms. Vera Molnar and Harold Cohen are examples of such computer artists [3–5]. Their output was limited by the algorithmic code used to derive geometric shapes and patterns, or line drawings, respectively. Recent research interest in robotic painting, such as e-David [6], FRIDA [7] and "Paul"[1] has employed advanced computer vision and graphics to enable closed-loop visual feedback systems.

However, from the perspective of craftsmanship, the utilization of machine-based operations poses a significant artistic constraint, as they are unable to capture the influence of the interaction between materials such as brushes, paint and canvas, that shapes the artistic process in a continuous interplay where the artist responds and adapts to the behaviour of materials.

This emphasis on responsiveness and interaction with materials is at odds with the rigid optimization frameworks of machine-based operations, highlighting the need for an intrinsically collaborative and interactive working process between humans and machines. In this vision, the machine not only responds to the visual state of the painting but also delves into the tactile realm of materials, extracting information from the brush and paper themselves. It then adapts its physical implementation of the following stroke accordingly.

Previous work in this direction, such as the show Incomputable Imagery: Reimagining the brushstroke[2] was, however, limited by specific code architectures [8–10].

It utilized algorithmic systems that generate brushstroke vectors, leveraging data extracted from the visual feedback mechanism. The iterative procedure starts with an initial "seed" stroke made by the artist. A snapshot of this stroke is taken by the camera and analyzed by a program. Using this data, a new stroke is constructed that traces the outlines, centre line and gradient of the input. At each step in this reiterative process, a different vision algorithm is used to

* These authors contributed equally to this work. Corresponding Authors emails: lioba.schuermann@t-online.de, giulia.dangelo@fel.cvut.cz

[1] a drawing robot by the artist Patrick Tresset http://patricktresset.com

[2] http://www.liatgrayver.com/projects/InComputable-Imagery-Reimagining-the-brushstroke (see figure 2 and http://www.liatgrayver.com/projects/Brushstrokes-in-the-Digital-Age

| Presynaptic population | Postsynaptic population | Synapse type | Tau (coarse,fine) value | Threshold (coarse,fine) value | Weight (coarse,fine) value |
|---|---|---|---|---|---|
| | | **P controller network** | | | |
| FPGA Spike generator | Theta inhibitory neurons | AMPA (fast excitatory) | 2,50 | 5,120 | 6,100 |
| FPGA Spike generator | Theta feedback neurons | AMPA (fast excitatory) | 2,50 | 5,120 | 6,100 |
| Theta feedback neurons | Theta feedback neurons | GABA A (fast inhbitory) | 2,25 | 6,240 | 6,240 |
| Theta neurons | Theta inhibitory neurons | GABA A (fast inhbitory) | 2,25 | 6,240 | 6,240 |
| Theta inhibitory neurons | Theta hidden neurons | GABA A (fast inhbitory) | 2,30 | 6,200 | 7,220 |
| Theta feedback neurons | Theta hidden neurons | AMPA (fast excitatory) | 2,50 | 5,150 | 6,150 |
| Theta hidden neurons | Theta error neurons | AMPA (fast excitatory) | 2,50 | 6,150 | 6,250 |
| Theta error neurons | Theta error neurons | GABA A (fast inhbitory) | 2,30 | 6,200 | 7,220 |
| | | **Inverse Kinematics Network** | | | |
| FPGA spike generator | Z inhibitory neurons | AMPA (fast excitatory) | 2,50 | 5,150 | 6,150 |
| Z neurons | Z neurons | NMDA (slow excitatory) | 2,50 | 4,150 | 5,160 |
| Z neurons | Z inhibitory neurons | GABA A (fast inhibitory) | 2,30 | 6,200 | 7,220 |
| Z inhibitory neurons | IK hidden neurons | GABA A (fast inhibitory) | 2,30 | 6,200 | 7,220 |
| State neurons | IK hidden neurons | AMPA (fast excitatory) | 2,50 | 5,150 | 6,150 |
| IK hidden neurons | Theta neurons | AMPA (fast excitatory) | 2,50 | 5,150 | 6,150 |
| | | **TDE Network** | | | |
| FPGA spike generator | Input neuron | AMPA (fast excitatory) | 3,60 | 5,180 | 5,150 |
| FPGA spike generator | Inhibitory neuron | AMPA (fast excitatory) | 3,60 | 5,180 | 5,150 |
| FPGA spike generator | Speed neurons | AMPA (fast excitatory) | 3,60 | 5,180 | 5,150 |
| Input neuron | Disinhibitory neuron | AMPA (fast excitatory) | 3,60 | 5,180 | 5,150 |
| Disinhibitory neuron | Inhibitory neuron | GABA A (fast inhibitory) | 2,22 | 5,150 | 5,160 |
| Inhibitory neuron | Output neurons | GABA A (fast inhibitory) | 2,60 | 4,200 | 5,120 |
| Input neuron | Delay neurons | AMPA (fast excitatory) | 3,70 | 4,190 | 5,200 |
| Input neuron | Delay neurons | GABA A (fast inhibitory) | 2,40 | 4,150 | 5,160 |
| Delay neurons | Delay neurons | AMPA (fast excitatory) | 3,70 | 4,190 | 5,200 |
| Delay neurons | Output neurons | AMPA (fast excitatory) | 3,40 | 5,150 | 5,100 |
| Output neurons | Speed neurons | AMPA (fast excitatory) | 3,60 | 5,180 | 5,150 |

Fig. 1: Extract of the employed synaptic parameters in the presented spiking network.

reconstruct a new image of the stroke and the e-David executes a new stroke that differs from the original due to the imperfections of the reconstruction procedure and the differing visual data extracted from each subsequent stroke in the process.

At regular intervals, the robot dips the brush into one of several ink containers, each containing ink diluted with a different amount of water. This procedure results in an ever-changing sequence of strokes that ultimately depend on a combination of variations in the amount and dilution of ink used by the robot and the reconstruction accuracy of the vision algorithm used at a given step.

The styles and techniques of operating the brush were predetermined, significantly restricting the genuine sense of collaboration between humans and machines within the system. As the only data that could be extracted to inform the system about the state of the stroke was visual, it was essentially impossible to plan a subsequent stroke that would intentionally overlap prior strokes. In existing computer vision techniques, intersecting marks are perceived by the system as a single, unified image. No information regarding the state of the paper, deformation of the brush or brushstroke velocity was available in the process. Gestural actions could not be designed, as the stroke was treated as static information, as an image. The system was essentially limited to merely planning the vectors of the next stroke to be executed.

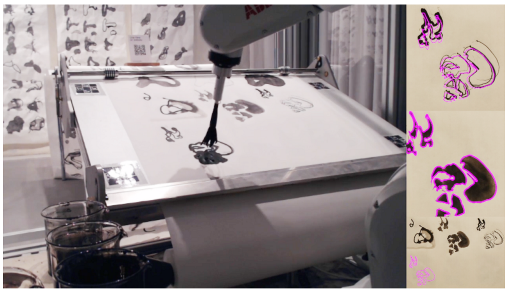

Fig. 2: Impression from the show Incomputable Imagery: Reimagining the Brushstrokes (2021). On the right is the visual feedback pipeline, generating the stroke's path based on the visual output of the previous stroke. Due to limitations in computer vision, overlapping strokes were not detectable.©VG Bild Kunst / Liat Grayver

The utilization of neuromorphic sensory-processing technology, a biology-inspired manner of implementing cognitive agents, presented an intriguing opportunity to delve into the realm of interactive robotics. This technology offers a real-time, dynamic interactive system that deviates from fixed algorithmic procedures and limited datasets, providing a more adaptive approach for exploring the art

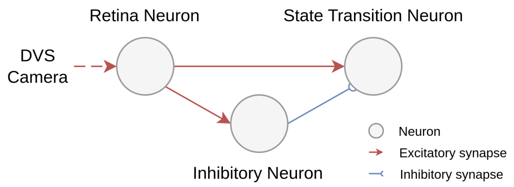


Fig. 3: Network architecture to determine a state transition in the visual scene. Each retina neuron excites an inhibitory and State Transition Neuron. The inhibitory neuron in turn suppresses the State Transition Neuron. Due to the delayed inhibition, the State Transition Neuron fires once whenever t he retina neuron is triggered anew.

of brushstrokes in the context of robotics-assisted painting. This is achieved through the integration of a Dynamic Vision Sensor (DVS) for visual input and a neuromorphic chip for sensory processing and pattern recognition, connected to a robotic arm with a force sensor. The focus of this exploration lies in the attempt to comprehend and replicate the essence of a single brushstroke.

The direct access to real-time dynamic data makes it possible to create and work with a new range of dynamic operations, which are at the core of the new work, Synaesthetic Strokes. This newfound capability has not only enhanced the machine's “understanding” of the intricate aspects required to recreate a stroke but has also allowed to assess the stroke's unfolding state, fostering a deeper level of physical collaboration akin to classical craftsmanship, where the affordances, impacts and potentialities of the human, medium and machine components comprising the system are intimately intertwined.

## III. Robotic setup

The following section provides a more in-depth description of the proposed network architecture and its characterization, which were utilized for the experiments presented in Section II.

### *A. Vision Module - Digital preprocessing and stroke detection*

On the computer, events are filtered to remove background noise, retain only the events generated by the light to dark change on the canvas due to the stroke (i.e. negative events) before subsampling the visual field. Subsampling is obtained by accumulating events from a receptive field of 30×20 pixels centred at the coordinate p(x,y) in Cartesian space. The receptive field with the highest event count, assuming a minimum activity of 10 events, triggers an event sent to the DYNAP-SE processor. The neurons receiving the events on the processor are named "retina neurons", where each neuron represents one subsampled coordinate p(x,y) in the visual field of the camera.

When a stroke ends, no further visual state transitions are generated for at least one second. The precise spike times can be extracted using the state transition network shown in Fig. 3 that employs delayed inhibition to signal the transition to a new visual state. The retina neuron population is connected via one-to-one excitatory connections to a population of State Transition Neurons. At the same

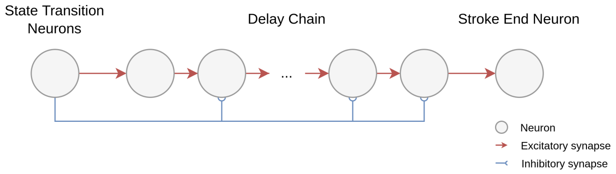


Fig. 4: Delay chain architecture to determine the end of a stroke. Each State Transition Neuron reinitiates a delay chain. The chain's last neuron triggers the stroke end neuron, indicating that the end of a stroke has been perceived.

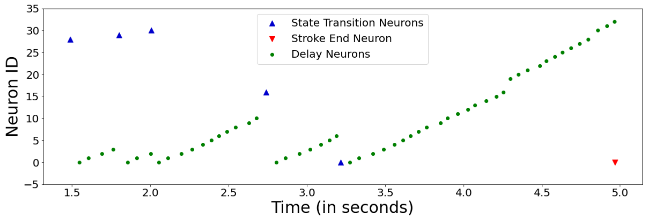


Fig. 5: Measurements of the State Transition, Delay and Stroke End neurons. Their activity determines whether a brushstroke detected in the visual scene has ended. Each State Transition Neuron (blue triangles), indicating the visual input, reinitiates a delay chain (green dots). The last neuron of the delay chain triggers the Stroke End neuron (red triangle). This is the case only if there has not been a state transition for at least as long as it takes a spike to propagate along the delay chain. The presented chain introduces a delay of approximately 1.5 seconds.

time, the retina neurons excite a population of interneurons that inhibit the state transition population in a one-to-one manner. The delayed inhibition causes the State Transition Neurons to fire once the visual input has transitioned to a new state and to remain silent afterwards.

The delay chain (see Fig. **??**) is responsible for detecting the end of a stroke. To achieve this, it assumes that a stroke has concluded if there has been no state transition for at least one second. Whenever a stroke transition happens, i.e. whenever one of the State Transition Neurons is activated, the first neuron of the delay chain is triggered, initiating a propagation along the chain. The spike signal travels from the first neuron to the Stroke End Neuron, taking on average  1 second. Every stroke transition additionally triggers an inhibition of the delay chain which occurs exclusively from the second neuron of the chain to the Stroke End Neuron. If no State Transition events occur during the signal's journey from the first delay chain neuron to the Stroke End Neuron, the network identifies the conclusion of the stroke. However, if one of the State Transition Neurons is triggered while the previous signal is propagating through the delay chain, the signal is inhibited, preventing the spike propagation to reach the Stroke End Neuron and consequently indicating that the stroke has not ended.

### *Experimental Results*

Figure 5 shows a raster plot of the state transition, delay and stroke-end neuron populations. The visualized activity was recorded on the DYNAP-SE chip during a painting trial. The firing times of the State Transition Neurons

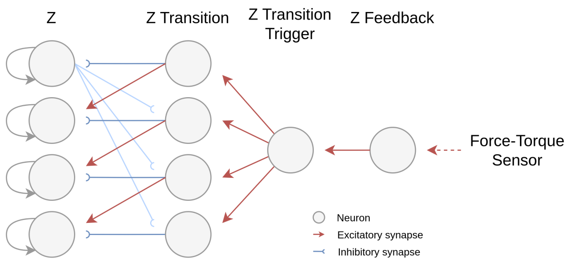


Fig. 6: Network architecture to update the desired z value. Each z neuron inhibits all z transition neurons except the one at the same index. Consequently, only a single neuron of the transition population can get triggered by external stimuli. Each z transition neuron suppresses the z neuron at the same index and excites the z neuron representing a higher or lower z value, leading to an adjustment of the desired z value.

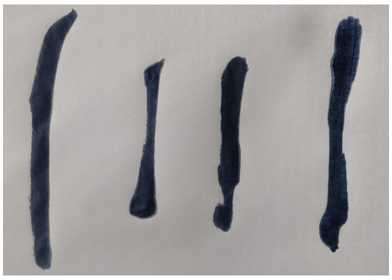

Fig. 7: Example brushstrokes generated by the spiking controller with integrated FT feedback.

signify the instances when the brushstroke transitions to a new visual state, as captured by the DVS camera. These neurons exhibit excitatory connections to the first neuron of the delay chain, while concurrently inhibiting all other neurons in the chain. Consequently, the activity of the State Transition Neurons triggers again the delay chain as can be seen on five occasions at times 1.5, 1.8, 2.0, 2.7 and 3.2 seconds in the plot. The spike-wave can only propagate along the whole delay chain in the absence of a new state transition, ultimately stimulating the stroke-end neuron. This event, occurring at approximately 5.0 seconds in the raster plot, serves as an indicator to the network that the detected brushstroke has ended. The activation of this neuron signals whether contact with the paper is desired or not.

## IV. Tactile Module

The force module is responsible for dynamically adjusting the end-effector's desired $z$ value to ensure constant contact of the brush with the paper. The employed Force-Torque sensor is a digital sensor and therefore requires an initial conversion of the acquired sensor values into a spike-based representation. Owing to the bendability of the brush bristles, the force values along the $z$-axis were deemed unsuitable for a meaningful analysis of the detected pressure and instead, the applied torque values along the $x$ and $y$ axes during the generation of a brushstroke are employed as more reliable indicators. In the first step, the measured torque values are post-processed on the computer-in-the-loop, consisting of the application of a fourth-order Butterworth filter smoothing the accumulated sensor data. Therefore, removing any bias introduced by the sensor and finally computing the average absolute value. For safety purposes, immediate removal of the brush from the paper is triggered if torque exceeds 0.25 Nm. From trial and error empirical measures, a range of torque values along the $x$ or $y$ axis corresponding to the acceptable pressure level on the paper was found to be [0.04, 0.12] Nm.

The measured pressure level is transmitted to the spiking controller on the neuromorphic processor via a population of feedback neurons. These neurons are encoded in a one-hot manner, with each neuron representing one of the possible pressure states: small, large, or within an acceptable pressure, to adjust the pressure of the brush onto the painting. Based on the computed pressure level, the processor's FPGA stimulates the corresponding neuron on the chip. The spiking neural network then adjusts the desired $z$ value of the controller. The network architecture responsible for updating the desired $z$ value is visualized in Fig. 6. Figure 6 only visualizes the update procedure of one of the three (smaller, large & acceptable) Z Feedback neurons. The $z$ population acts as a memory unit and represents the network's current $z$ value, implemented as a state machine, and characterized by a single active neuron.

Each $z$ neuron inhibits all $z$ transition neurons except the one at the same index. The $z$-population additionally receives excitatory input from the $z$ transition trigger neuron which is activated by one of the $z$ feedback neurons. Due to the inhibitory connections, only a single neuron of the transition population can get triggered by external stimuli. Each $z$ transition neuron suppresses the z neuron at the same index and excites the $z$ neuron representing a higher or lower $z$ value, leading to an adjustment of the desired $z$ value.

To illustrate the impact of tactile feedback on the painting process, a series of exemplary brushstrokes is presented in Fig. 7. For simplicity, the robotic controller was instructed to generate a collection of straight lines, each executed from top to bottom. In particular, the second stroke from the right highlights the impact of the integrated pressure feedback. Towards the lower end of the strokes, a decrease of applied pressure occurs, due to different factors such as brush deformation or ending the stroke, resulting in a thinner line being painted. This reduction in pressure is detected by the system and leads to an adjustment of the $z$ value (See Figure 5). The adjustment of the $z$ value ensures consistent contact with the paper surface, leading to a visibly widened stroke compared to the stroke before any pressure adjustment.

## V. Motor Module

The motor module computes a series of joint velocity commands for the robotic end-effector to follow a desired trajectory. Due to the heterogeneous nature of the spiking neural network, trajectories are not replicated identically. To illustrate the resulting variance, Figure 8 compares the trajectory of the robot's end-effector along the $x$ and $y$ axes as it follows the same path across five trial runs. The $z$-axis value remained constant throughout the trials. The

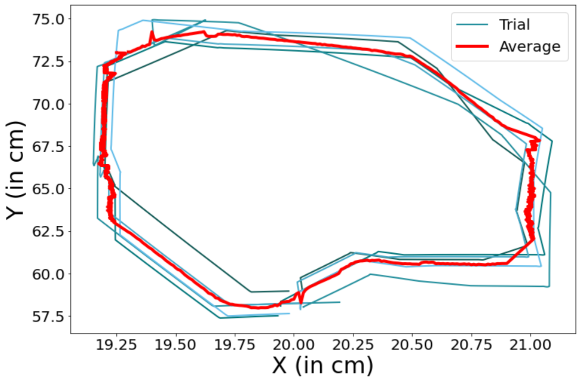


Fig. 8: Comparison of the trajectory of the robot's end-effector along the x and y axes across five trial runs with the same prescribed path. The trial trajectories are depicted in blue and the averaged trajectory across the trials is shown in red.

individual trial trajectories are visualized in blue, while the average trajectory across all trials is shown in red. The individual trajectories exhibit a variance of up to ~1 cm along the $x$ and $y$ axes when compared to the average trajectory.

## References


[1] P. Livi and G. Indiveri, "A current-mode conductance-based silicon neuron for address-event neuromorphic systems," in *2009 IEEE International Symposium on Circuits and Systems*, 2009, pp. 2898–2901. DOI: 10.1109/ISCAS.2009.5118408.

[2] M. McLuhan, *The Medium Is the Massage: An Inventory of Effects*. Bantam Books, 1967.

[3] A. Guillermet, "Vera molnar's computer paintings," *Representations*, vol. 149, pp. 1–30, Feb. 2020. DOI: 10.1525/rep.2020.149.1.1.

[4] L. Sundararajan, "Harold cohen and aaron: Collaborations in the last six years (2010–2016) of a creative life," *Leonardo*, vol. 54, pp. 1–10, Mar. 2020. DOI: 10.1162/leon_a_01906.

[5] P. Cohen, "Harold cohen and aaron," *AI Magazine*, vol. 37, no. 4, pp. 63–66, Jan. 2017. DOI: 10.1609/aimag.v37i4.2695. [Online]. Available: https://ojs.aaai.org/aimagazine/index.php/aimagazine/article/view/2695.

[6] T. Lindemeier, "E-david : Non-photorealistic rendering using a robot and visual feedback," Ph.D. dissertation, Universität Konstanz, Konstanz, 2018.

[7] P. Schaldenbrand, J. McCann, and J. Oh, *FRIDA: A Collaborative Robot Painter with a Differentiable, Real2Sim2Real Planning Environment*, arXiv:2210.00664 [cs], Oct. 2022. [Online]. Available: http://arxiv.org/abs/2210.00664 (visited on 07/21/2023).

[8] D. Berio, F. F. Leymarie, P. Asente, and J. Echevarria, "Strokestyles: Stroke-based segmentation and stylization of fonts," *ACM Trans. Graph.*, vol. 41, no. 3, Apr. 2022, ISSN: 0730-0301. DOI: 10.1145/3505246. [Online]. Available: https://doi.org/10.1145/3505246.

[9] T. Lindemeier, M. Spicker, and O. Deussen, "Artistic Composition for Painterly Rendering," in *Vision, Modeling & Visualization*, M. Hullin, M. Stamminger, and T. Weinkauf, Eds., The Eurographics Association, 2016, ISBN: 978-3-03868-025-3. DOI: 10.2312/vmv.20161350.

[10] J. Gülzow, L. Grayver, and O. Deussen, "Self-improving robotic brushstroke replication," *Arts*, vol. 7, no. 4, p. 84, Nov. 2018, ISSN: 2076-0752. DOI: 10.3390/arts7040084. [Online]. Available: http://dx.doi.org/10.3390/arts7040084.
